# Disentangling drivers of cross-domain microbial β-variations in intertidal mudflats

**DOI:** 10.1128/msystems.01777-25

**Published:** 2026-02-26

**Authors:** Xiaofan Gong, Xia Liu, Kai Ma, Jiayin Zhou, Wen Song, Yueyue Li, Qichao Tu

**Affiliations:** 1 Institute of Marine Science and Technology, Shandong University 520252, Qingdao, China; 2 Qingdao Key Laboratory of Ocean Carbon Sequestration and Negative Emission Technology, Shandong University 520252, Qingdao, China; 3 Southern Marine Science and Engineering Guangdong Laboratory (Zhuhai), Zhuhai, China; University of Memphis, Memphis, Tennessee, USA

**Keywords:** mudflat intertides, latitudinal β-variations, microbial domains, community assembly, regional species pool, gamma diversity, environmental heterogeneity

## Abstract

Understanding how different microbial groups respond to broad environmental gradients is essential for revealing the processes that structure microbial diversity. In this study, we comparatively investigate the ecological drivers that shape β-diversity across different microbial domains/kingdoms (bacteria, archaea, fungi, and protists) in intertidal mudflats along a broad climatic gradient, spanning ~18,000 km along the Chinese coastline. Distinct latitudinal β-diversity patterns are observed for different microbial domains/kingdoms. Null model analyses reveal significant deviations in β-diversity from null expectations across all microbial domains, with bacterial and archaeal β-diversity primarily associated with the γ-diversity or regional species pools, whereas fungal and protist communities were more strongly shaped by local community assembly processes. Homogeneous selection is the predominant assembly process, with varied relative influence of environmental variables across different domains/kingdoms as revealed by db-RDA. These findings underscore the importance of domain-specific ecological processes in shaping microbial biogeography and highlight the need for comparative frameworks across microbial groups to understand biodiversity patterns along environmental gradients.

**IMPORTANCE:**

Understanding the spatial distribution of biodiversity is a fundamental goal in ecology, yet most microbial studies focus on single domains. This study provides a comprehensive comparison of bacteria, archaea, fungi, and protists along an ~18,000 km latitudinal gradient in intertidal mudflats. We reveal that these microbial domains do not follow a unified diversity pattern but are instead governed by distinct ecological drivers. Bacteria and archaea are strongly influenced by regional species pools, whereas fungal and protist communities are primarily shaped by local stochastic processes such as dispersal limitation. These findings highlight the importance of organismal traits (e.g., body size) in shaping community assembly. This work emphasizes the necessity of establishing a multi-domain framework to accurately predict how Earth’s complex microbiomes respond to environmental changes.

## INTRODUCTION

Understanding biodiversity patterns across geographic gradients and the underlying ecological drivers that shape them is a key topic in ecology (1, 2). One of the most important patterns in ecology is the latitudinal diversity gradient (LDG), which describes the pattern of decreasing biodiversity with increasing latitude (3, 4). Over the past years, advancements in molecular ecology have revealed that microbial communities, often referred to as the “unseen majority” (5), also exhibit typical latitudinal diversity gradient patterns as macroorganisms, though exceptions have also been reported (6, 7). In general, microorganisms exhibit a less pronounced latitudinal diversity gradient compared to macroorganisms (8, 9). This shall be attributed to the unique biological traits possessed by microorganisms, such as small body size, high dispersal ability (both active and passive), high functional redundancy, and horizontal gene transfer (10–12), which may obscure or modulate traditional latitudinal diversity patterns. Therefore, understanding the ecological drivers of microbial biodiversity across broad environmental gradients is critical to advancing microbial biogeography and ecology.

Biodiversity encompasses multiple types of measurements across spatial scales, namely α-, β-, and γ-diversity, respectively, representing the diversity at local, between-site, and regional scales. Despite extensive research on the LDG of macro- and microorganisms, the variations in microbial β-diversity and the ecological drivers underneath remain understudied, with conflicting reports on latitudinal trends among microbial domains/kingdoms. Mechanically, both regional species pools and local community assembly may shape the patterns of β-diversity (13–16). In the first scenario, a large regional species pool allows more species to colonize the habitat, resulting in stronger biological interactions and greater β-diversity. Variations in β-diversity across broad biogeographic gradients are more likely to be driven by γ-diversity than by local community assembly (13). In the second scenario, local community assembly processes also contribute to the variations in β-diversity. Among these processes, heterogeneous selection filters species from the regional species pools based on their niche preferences, leading to dissimilar community composition and high β-diversity (16, 17). In contrast, homogeneous selection results in convergent communities and thereby low β-diversity (18). In addition, demographic processes, such as dispersal, speciation, and extinction, also affect β-diversity (19, 20). Specifically, homogeneous dispersal, defined as the unrestricted and uniform movement of individuals across sites, leads to low turnover among communities, whereas dispersal limitation increases community heterogeneity across sites (20, 21). Over the past years, contrasting mechanisms have been reported to explain the latitudinal variation of β-diversity for macrobes and microbes (13, 15, 22).

However, it should be noted that the differences among different microbial domains/kingdoms (e.g., bacteria, archaea, fungi, and protists) could be as large as those between macrobes and microbes (23, 24). The complex microbial world is composed of multiple domains/kingdoms, such as bacteria, archaea, fungi, protists, and the more diverse viruses, of which fungi and protists are different kingdoms in the three-domain system. Distinct microbial domains/kingdoms exhibit remarkable differences in their specific organismal traits, particularly body size, a fundamental attribute that significantly influences key ecological characteristics such as total abundance, growth rate, metabolic activity, and dispersal potential (25–27). Recent studies on microbial communities report that body size plays a significant role in shaping ecological patterns and processes (25, 28). Therefore, resolving the ecological patterns and their underlying drivers for different microbial domains is expected to provide valuable insights into how the complex unseen majority is composed in the Earth’s biosphere.

The coastal mudflat intertidal wetland is a land-sea interface that is strongly and frequently influenced by various geological, hydrological, physicochemical, and biological factors (29). Consequently, microbial communities in the intertidal wetlands are associated with unique physiological and genetic characteristics and are representative as a model dynamic system for microbial ecological studies (30, 31). In this study, the ecological drivers of latitudinal β-variations across different microbial domains/kingdoms were comparatively investigated in intertidal sediments along the Chinese coasts, spanning from the southmost (Sanya) to the northmost (Dandong) coastal regions. The following ecological questions were addressed in this study: (i) How do different microbial domains/kingdoms differ in β-diversity patterns along a latitudinal gradient? (ii) What are the ecological mechanisms driving the latitudinal β-variations of different microbial domains/kingdoms? We expected dramatically different β-variation patterns for different microbial domains/kingdoms, primarily due to their different lifestyles, behavioral capabilities, and metabolic activities. Consequently, we hypothesized that different ecological processes drove the domain-level differentiation of latitudinal β-variation patterns. This study aims to provide important ecological insights into the mechanisms shaping the β-variations across different microbial domains/kingdoms along a broad climate gradient.

## MATERIALS AND METHODS

### Sampling sites and collection of intertidal sediments

**Fig 1:**
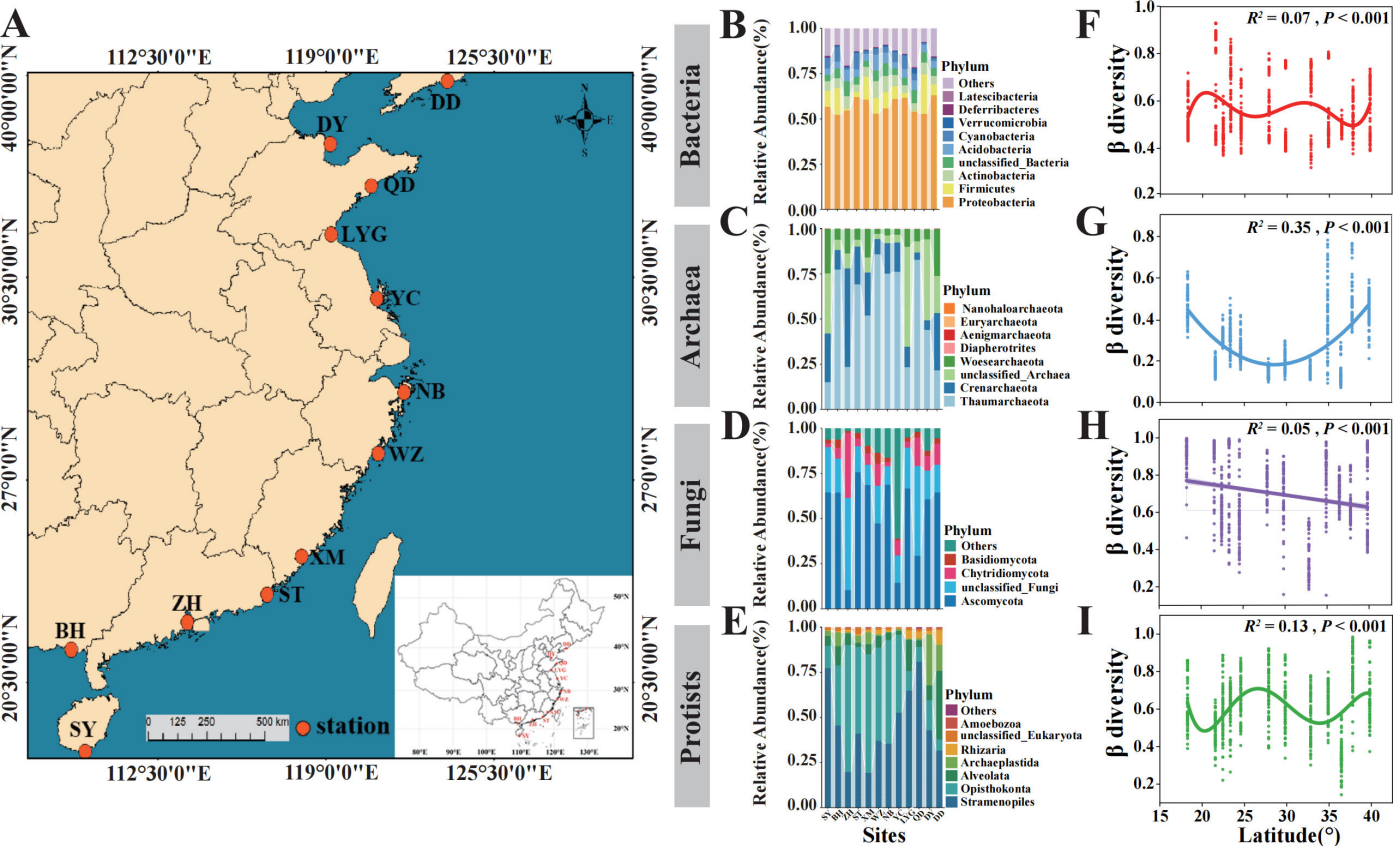
Sampling sites and overall microbial diversity along latitudes in the intertidal zones. (**A**) Geographic locations of the 12 sampled mudflat intertidal zones along the Chinese coast, including Sanya (SY), Beihai (BH), Zhuhai (ZH), Shantou (ST), Xiamen (XM), Wenzhou (WZ), Ningbo (NB), Yancheng (YC), Lianyungang (LYG), Qingdao (QD), Dongying (DY), and Dandong (DD). Orange circles represent the sampling sites. (**B–E**) Phylum-level composition of four different microbial domains, including bacteria, archaea, fungi, and protists. (**F–I**) The β-diversity patterns of different microbial domains along the latitudinal gradient. The pairwise community dissimilarity (Bray-Curtis) among different samples in each site represents β-diversity.

In this study, intertidal sediment samples were collected from 12 mudflat intertidal regions along the Chinese coasts, which spanned from the southernmost (Sanya, 18°27′ N) to the northernmost (Dandong, 39°81′ N) coastal regions in China, and from 109°15′ E to 123°69′ E, covering approximately 18,000 km of the coast line (Fig. 1A; Table S1). To minimize the influence of seasonal variation and balance temperature differences across latitudes, sampling was conducted sequentially from south to north between April (Sanya) and June 2021 (Dandong), with some adjustments in Shantou and Wenzhou due to local weather conditions (Table S1). At each site, samples were collected during low tide within an area of 1000 m × 200 m (Fig. S1), when the intertidal sediment was fully exposed. A total of 180 sediment samples were obtained, with 15 samples per region (Fig. S1). At each sampling point, five surface sediment cores (~15 cm deep) were collected within a 1 m² area and homogenized to form a composite sample. The sediment samples were then packed in sterilized polyethylene bags and stored on ice before being transported to the laboratory. Approximately 200 g of sediment was retained for each sample, of which 100 g was kept at 4°C for physicochemical analysis, and the remaining 100 g of sediment was freeze-dried and stored at −80°C for DNA extraction.

### Environmental parameter measurements

A total of 11 physicochemical variables were measured for the sediment samples, including pH, temperature, salinity, moisture content, ammonia-nitrogen (NH_4_^+^-N), nitrate-nitrogen (NO_3_^−^-N), nitrite nitrogen (NO_2_-N), total nitrogen (TN), total phosphorus (TP), water-soluble sulfate (SO_4_^2−^), and total organic carbon (TOC). The soil pH was measured using a pH meter (STARTER 300, OHAUS, Beijing, China) with a soil-to-water ratio of 1:5 (wt/vol). The temperature was measured *in situ* using a mercury thermometer (−50°C to 50°C) during sample collection. The salinity was measured using a standard method using a salinity meter (WS-31, Xudu, Beijing, China) (32). The soil moisture was measured by the weight difference after freeze-drying 20 g of soil until a constant weight was reached. The concentration of SO_4_^2−^ was determined by barium chromate spectrophotometry. The concentrations of NH_4_^+^-N, NO_2_^−^-N, and NO_3_^−^-N were measured by KCl solution extraction-spectrophotometry at 630 nm, 543 nm, and 410 nm, respectively (33, 34). TN was measured by alkaline potassium persulfate digestion and UV spectrophotometry at 410 nm (35). TP was determined by ammonium molybdate spectrophotometry at 700 nm to determine the absorbance value (34). All absorbance readings were performed using a microplate reader (Cytation5, BioTek Instruments, USA). The TOC was quantified using the dichromate digestion method with a TOC analyzer (TOC-L CPH, Shimadzu, Kyoto, Japan) (36). The values of all measured environmental variables are provided in Table S2.

### DNA extraction, amplification, and sequencing

Total sediment DNA was extracted from 0.25 g dried sediment samples using the FastDNA SPIN Kit for Soil (MP Biomedicals, Solon, USA). The DNA was purified using the DNA Clean & Concentrator−100 kit (Zymo Research) following the manufacturer’s instructions. The concentration and quality of the extracted DNA were determined using a NanoDrop ND-250 spectrophotometer (NanoDrop Technologies Inc., Wilmington, DE). Different microbial domains were profiled by targeting specific regions of the genetic markers for bacteria, archaea, fungi, and protists. The following regions of phylogenetic markers were targeted: V4-V5 region of the 16S rRNA gene for bacteria, V4-V5 region of 16S rRNA for archaea, the internal transcribed spacer 2 (ITS2) region for fungi, and the 18S rRNA V4 region for protists. Polymerase chain reaction (PCR) assays were performed using universal primer pairs for each genetic marker. The primer pairs were 338F/806R (338F, 5′-ACTCCTACGGGAGGCAGCA-3′; 806R, 5′- (GGACTACHVGGGTWTCTAAT-3′) for bacteria (37, 38), 524F-10-ext/Arch958R-mod (524F-10-ext, 5′-TGYCAGCCGCCGCGGTAA-3′ Arch958R-mod, 5′-CCGGCGTTGAVTCCAATT-3′) for archaea (39), gITS-7F/ITS-4R (gITS-7F, 5′-GTGARTCATCGARTCTTTG-3′; ITS-4R, 5′- TCCTCCGCTTATTGATATGC-3′) for fungi (40), and 528F/706R (528F, 5′-GCGGTAATTCCAGCTCCAA-3′; 706R 5′-AATCCRAGAATTTCACCTCT-3′) for protists (41). PCR amplification was performed in a 50 μL volume, including 25 μL 2× Premix Taq (Takara Biotechnology, Dalian Co. Ltd., China), 1 μL each primer (10 μM), and 3 μL DNA (20 ng/μL) template. The conditions for PCR amplification included 30 cycles of denaturation at 94°C for 30 s, annealing at 55°C for 45 s, and extension at 72°C for 45 s, and a final extension at 72°C for 5 min. PCR products were then paired-end sequenced on the Illumina NovaSeq 6000 platform (paired-end, 2 × 150 bp, Inc., San Diego, CA, USA) at Novogene Bioinformatics Technology Co. Ltd. (Beijing, China). All 180 samples underwent DNA extraction and sequencing. Based on sequencing depth and data quality, 12 high-quality samples from each site were retained for microbial community analysis, yielding a final data set of 144 samples used in the current study.

### Processing of sequencing data

Raw sequencing data processing, including quality filtering, sample inference, forward-reverse merging of paired reads, deduplication, and chimera identification, was carried out using the Divisive Amplicon Denoising Algorithm 2 (DADA2) package, which is a model-based approach for correcting Illumina amplicon errors, eliminating the need to build operational taxonomic units (OTUs) (42). Taxonomic assignments of amplicon sequence variants (ASVs) were done using the Ribosomal Database Project (RDP) database (https://github.com/rdpstaff/classifier) (43) for bacteria and archaea, the UNITE database (version 7.2) (44) for fungi, and the Protist Ribosomal Reference database (PR^2^) (version 5.0.1) (45) for protists. In addition, we excluded ASVs identified as eukaryotes, mitochondria, and chloroplasts from the bacterial ASV table, non-archaeal ASVs from the archaeal ASV table, non-fungal ASVs from the fungal ASV table, and fungi, plants, and animals from the protists ASV table. For fungal ASVs, sequences were searched against the NCBI database (46) using BLAST (Basic Local Alignment Search Tool) (47), and non-fungal sequences were removed based on the taxonomic information assigned by the MEGAN software (48). Finally, the taxonomic information of fungal ASVs was assigned using the UNITE database (44). To ensure homogeneity of all sequenced samples, the sequencing data were rarefied using the “rarefy” function of the “GUniFrac” package (49).

### Statistical analyses

All statistical analyses were conducted in R (version 4.3.1). The α-diversity was defined as the observed richness of ASVs for each of the 12 samples in each sampling region and was calculated using the “vegan” package. The β-diversity was defined as the community dissimilarity between different samples. The Bray–Curtis dissimilarities were calculated among all 144 samples to characterize between-sample compositional variations of microbial communities. To quantify β-diversity at the site level, we computed the mean pairwise Bray–Curtis dissimilarity among the 12 samples within each region, representing intra-site compositional variations. To evaluate whether β-diversity significantly varied among the 12 sites, Kruskal–Wallis tests were performed using the “kruskal.test” function in the “stats” package. The “vegdist” function in the “vegan” package (50) was used to calculate Bray–Curtis dissimilarity. The γ-diversity was defined as the total ASV richness of the 12 samples from each sampling region and was calculated using the “specpool” function in the “vegan” package. The relationships between microbial diversity (α, β, and γ) and latitudes were evaluated using linear and polynomial regression models. The 11 environmental factors were standardized using the “scale” function in R to reduce the effect of numerical magnitude between environmental factors. The Euclidean distance was calculated based on the standardized environmental variables as a measurement of environmental heterogeneity. Distance-based redundancy analysis (db-RDA) was performed using the “capscale” function in the “vegan” package to evaluate the relationships between microbial community composition and environmental factors (51).

To test the impact of γ-diversity on β-variations, we first examined the expected relationship between β-diversity and γ-diversity in the absence of any community assembly process other than random sampling. Specifically, we first employed the “taxo.null” function from the “NST” package in R, which generates expected null communities by processing species occurrence frequencies (sp.freq), species richness in each sample (samp.rich), and relative abundance (abundance). Subsequently, we calculated the relationship between β-diversity and γ-diversity of the expected null communities. We then evaluated the relationship between the observed β-diversity and γ-diversity through linear regression models. If the variations in β-diversity were mainly driven by γ-diversity, the expected relationship between β-diversity and γ-diversity should be similar to the observed one.

To investigate the impacts of regional species pools and local community assembly processes on β-diversity, two different types of null models were generated by utilizing the “taxo.null” function of the “NST” package (52). In model I, the species pool was solely considered as the number of observed species within a region, that is, γ-diversity. In model II, the species pool was considered as the observed number of species and the relative abundance of species within a region. For each model, expected β-diversity was generated from 1,000 permutations, and β-deviation was calculated as follows:


(1) $$\beta_{deviation}=\frac{\beta_{obs}-\beta_{null}}{SD\left(\beta_{null}\right)}$$

A positive mean β-deviation indicates that β-diversity is primarily driven by heterogeneous selection (HeS) or dispersal limitation (DL), whereas a negative mean β-deviation reflects the influence of homogeneous selection (HoS) or homogenizing dispersal (HD). A mean β-deviation of zero indicates that stochastic processes, such as drift, dominate the variation in β-diversity. A schematic overview of the null model workflow and its ecological interpretation is illustrated in Fig. S6.

To gain further insight into the relative contributions of different local community assembly processes, the null model analysis approach based on β-nearest taxon index (βNTI) and Raup-Crick metric (RC) was employed (20). The fraction of pairwise comparisons with βNTI < −2 was considered as the percentage of homogeneous selection, while the fraction of pairwise comparisons with βNTI > 2 was the percentage of heterogeneous selection. Next, the RC metric was used to partition the remaining pairwise comparisons with |βNTI| ≤ 2. The fraction of pairwise comparisons with RC_bray_ < − 0.95 was treated as the percentage of homogenizing dispersal, while those with RC_bray_ > + 0.95 were treated as dispersal limitation and |RC_bray_| ≤ 0.95 as drift. The βNTI and RC_bray_ were calculated using the “picante” and “iCAMP” packages in R.

## RESULTS

### Overall intertidal microbial diversity patterns along the latitudes

After quality filtering and removal of chimeric sequences, a total of 3,029,253 bacterial, 2,232,288 archaeal, 963,594 fungal, and 4,235,472 protist sequences were obtained. These sequences were further resolved into 94,821 bacterial amplicon sequence variants (ASVs), 45,515 archaeal ASVs, 16,477 fungal ASVs, and 21,458 protist ASVs (Tables S3 and S4). For each sample and microbial domain, the number of sequences was rarefied to the same sequencing depth. The overall taxonomic composition of different microbial domains at the phylum level was generally similar to those in other similar ecosystems (Fig. 1B through E). For bacteria, Proteobacteria (57.15%) was the dominant phylum, followed by Firmicutes (7.99%), Actinobacteria (5.96%), and Acidobacteria (4.81%). While Proteobacteria maintained a relatively consistent proportion across sites, other phyla displayed notable variability along the latitudinal gradient. For archaea, Thaumarchaeota (53.78%) was the dominant phylum, followed by Crenarchaeota (19.24%) and Euryarchaeota (16.44%). The dominance of Thaumarchaeota was particularly pronounced in low- to mid-latitude sites (e.g., 18°, 22°, and 29°), where it consistently exceeded 70% of the community composition. However, its relative abundance declined slightly at high-latitude sites (e.g., 34° and 39°), where other phyla became more prominent. For fungi, Ascomycota (52.76%) was the most dominant phylum across all latitudes, maintaining a consistently high relative abundance in low-latitude regions (e.g., 18° and 22°), where it accounted for more than 70% of the fungal community. However, its dominance decreased gradually with increasing latitude, particularly in high-latitude sites (e.g., 36° and 39°), where other fungal phyla, such as Chytridiomycota (9.74%) and Basidiomycota (2.86%), became more prominent. For protists, the relative abundances of Stramenopiles and Opisthokonta were 45.59% and 35.39%, respectively. Generally, the protist community structure showed substantial shifts along the latitudinal gradient, with Stramenopiles dominating low-latitude regions and Opisthokonta being more prominent at higher latitudes.

Different microbial domains were found with contrasting latitudinal diversity patterns. The α- and γ-diversity of bacteria and archaea decreased with increasing latitude, while the ones of fungi increased with increasing latitude (Fig. S2A through C and S2E through G). Notably, no latitudinal pattern was observed for protists (*P* > 0.5) (Fig. S2D and H). For all microbial domains, the α- and γ-diversity were strongly and significantly associated (Fig. S2I through L). Unlike α- and γ-diversity, the variation in microbial β-diversity along latitude were generally curvilinear, except for fungi (Fig. 1F through I). For bacteria, the β-diversity fluctuated along latitude, with low values observed at 22° and 36° N, and high values at 21°, 23°, and 32° N (Fig. 1F). For archaea, the β-diversity was “U” shaped along latitude, with low values found at 27° and 36° N, whereas high values at 18°, 37°, and 39° N (Fig. 1G). For protists, the β-diversity also fluctuated along latitude, with low values at 21° and 36° N and high values at 27° and 37° N (Fig. 1H). For fungi, a decreasing pattern from low to high latitude could be observed (Fig. 1I). Statistical analysis using Kruskal–Wallis tests confirmed that β-diversity significantly differed among the 12 sites for all microbial domains (bacteria: χ² = 271.07, *P* < 0.001; archaea: χ² = 1246.9, *P* < 0.001; fungi: χ² = 704.71, *P* < 0.001; protists: χ² = 613.94, *P* < 0.001). The results suggested that different ecological processes may have driven the latitudinal β-variations of different microbial domains.

### Relationship between regional species pool and β-diversity

**Fig 2:**
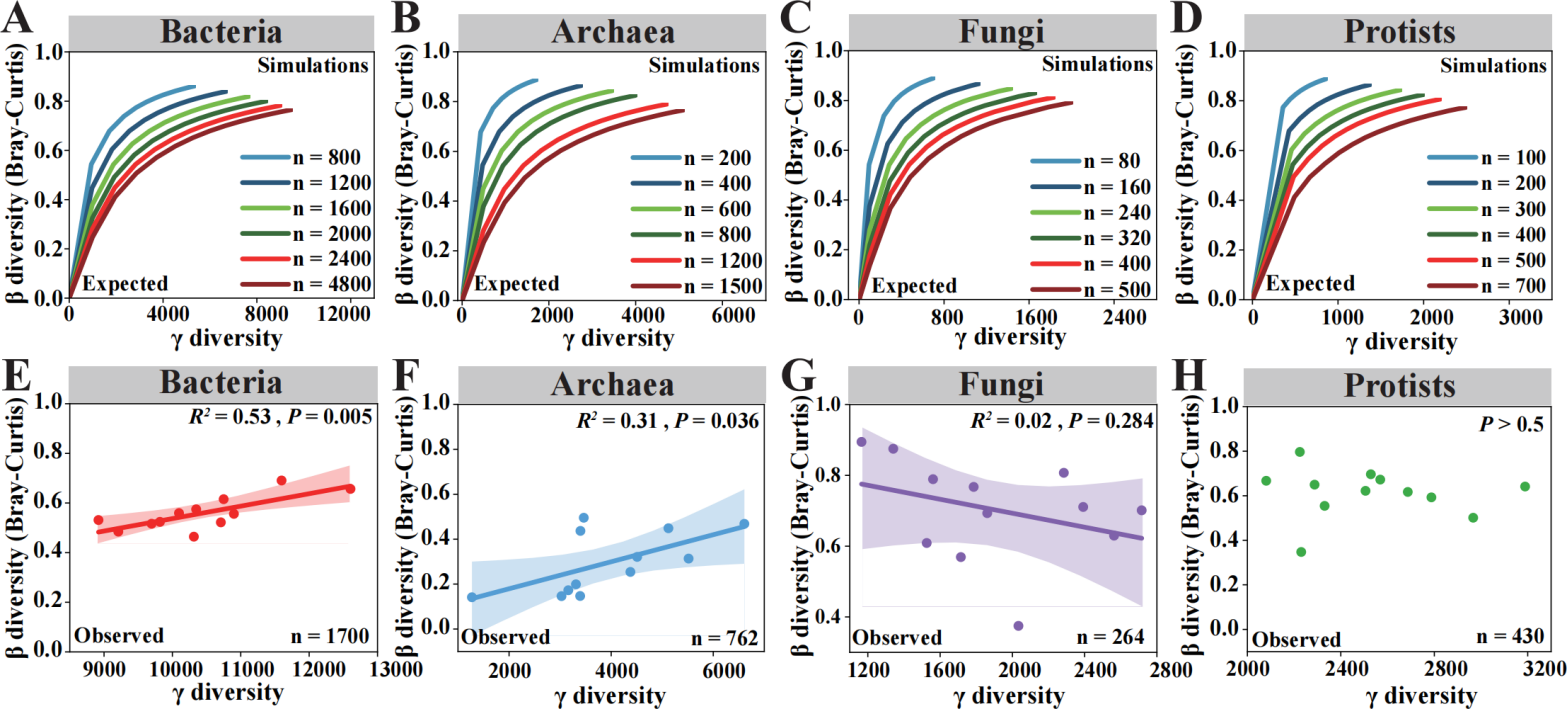
The relationships between β-diversity and γ-diversity of different microbial domains. (**A–D**) The expected algebraic relationships between β-diversity and γ-diversity are solely based on random sampling from the species pool. The lognormal species abundance distributions were followed for sampling. The *n* value represents the number of randomly sampled individuals. (**E–H**) The relationships between observed β-diversity and γ-diversity based on the empirical data of different microbial domains.

We first examined the relationship between γ- and β-diversity for different microbial domains by investigating their expected algebraic relationship in the absence of any community assembly process other than random sampling. For all microbial domains, the expected β-diversity increased with γ-diversity, regardless of the number of individuals involved (Fig. 2A through D). However, a divergent relationship was found between the observed β- and γ-diversity along the latitudes (Fig. 2E through H). For bacteria (*R^2^* = 0.53, *P* = 0.005) and archaea (*R^2^* = 0.36, *P* < 0.05), significant positive correlation was found between the observed β- and γ-diversity (Fig. 2E and F). In contrast, for fungi (*P* = 0.28) and protists (*P* > 0.5), no significant association was found between the observed β- and γ-diversity (Fig. 2G and H).

**Fig 3:**
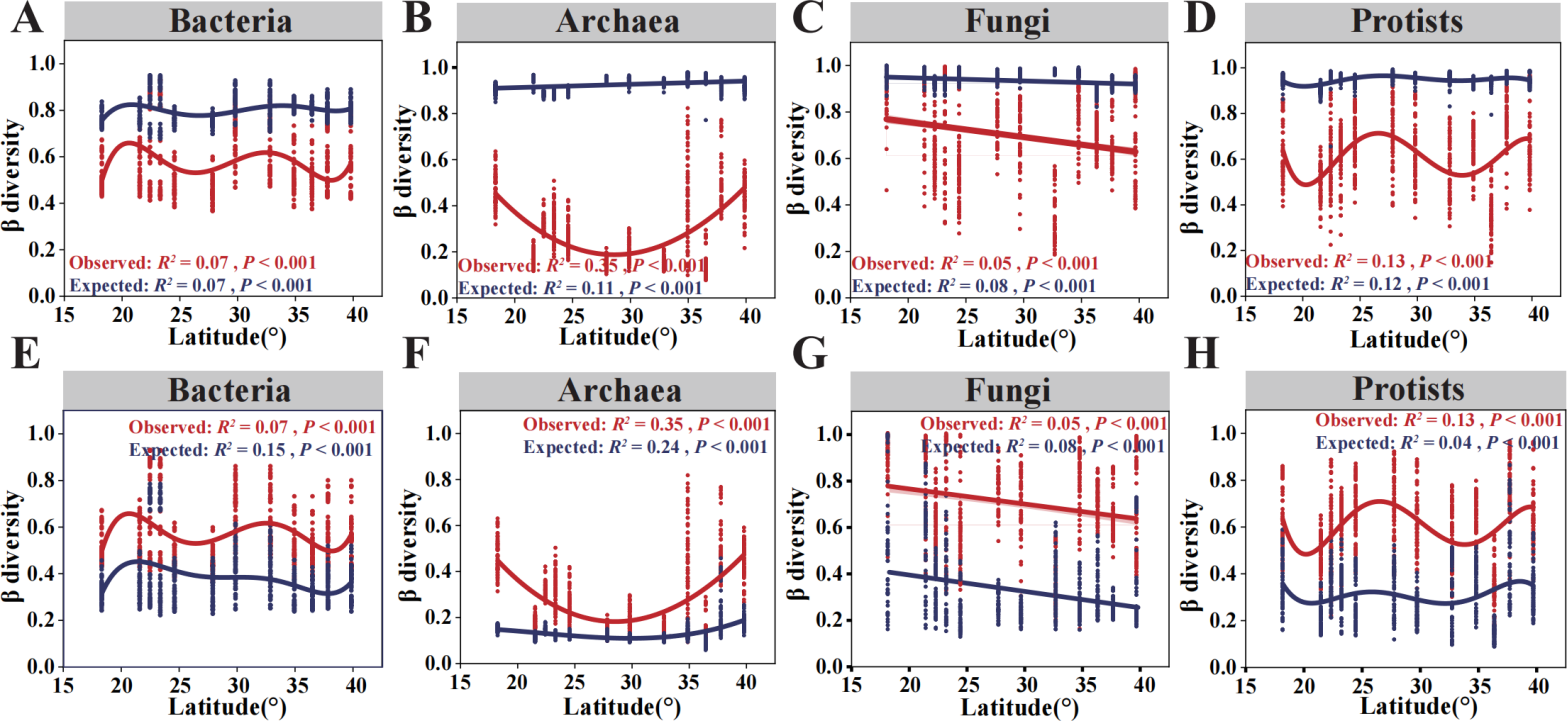
Null model simulations of β-diversity patterns for different microbial domains along the latitudinal gradient. (**A–D**) The observed (red) and expected (blue) β-diversity based on Bray–Curtis dissimilarity using null models controlling for γ-diversity. (**E–H**) The observed (red) and expected (blue) β-diversity based on Bray–Curtis dissimilarity using null models controlling for regional species pool. From left to right are bacteria, archaea, fungi, and protists.

Then, we examined whether and how the observed β-diversity deviated from the null expectations under different constraints, either by controlling γ-diversity or the regional species pool. In either case, the observed β-diversity significantly deviated from the null expectations, and the directions of deviations depended on the constraints in generating null models (Fig. 3). When the null models were generated by controlling γ-diversity, the observed β-diversity of all microbial domains was generally smaller than the null expectations (Fig. 3A through D). In contrast, the observed β-diversity of all microbial domains was higher than null expectations when null models were generated by controlling the regional species pool (Fig. 3E through H). Notably, clear inconsistency could be observed between the expected and observed β-diversity patterns of archaea along the latitudes (Fig. 3B and F), indicating that regional species pool other than local community assembly may have dominated the β-diversity variations of the archaeal assemblages. Such results suggested that the β-diversity of intertidal microbial communities was simultaneously and differently shaped by multiple ecological drivers (e.g., regional species pool, deterministic and stochastic processes).

### Ecological processes shaping the β-diversity of different microbial domains

**Fig 4:**
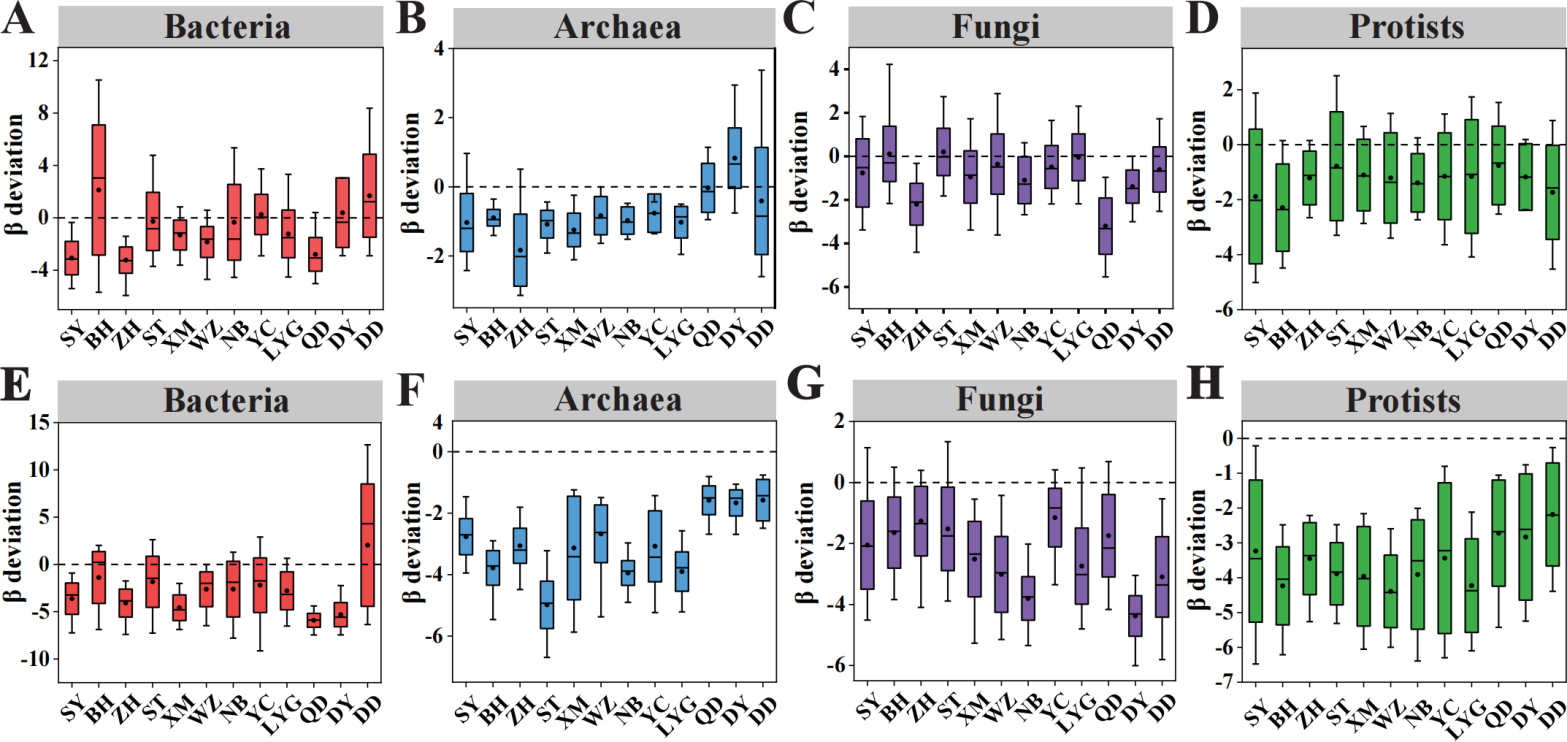
Patterns of β-deviations of different microbial domains along the latitudinal gradient. (**A–D**) The β-deviations along latitudes were assessed using null models controlling for γ-diversity. (**E–H**) The β-deviations along latitudes were assessed using null models controlling for the regional species pool. The β-deviation is the standardized effect size of β-diversity. The bottom and top of each box represent the first and third quartiles, the inner line represents the median, and the inner dot represents the mean.

Multiple statistical testing and analyses were conducted to disentangle the underlying ecological processes shaping the β-diversity of different microbial domains. First, the patterns of β-deviations (the deviation of β-diversity from null expectations) along the latitudes were examined for different microbial domains, by controlling the differences in γ-diversity (Fig. 4A through D) and the regional species pool (Fig. 4E through H). In either case, the patterns of β-deviations along latitudes were generally similar to those of β-diversity, except for archaea (Fig. 4B and F). The mean β-deviations of all microbial domains were generally smaller than zero, except for a few locations (e.g., bacterial assemblages at 21°, 32°, 37°, and 39° N; archaeal assemblages at 37° N) (Fig. 4A and B). This suggested that homogeneous selection or homogeneous dispersal tended to be the major local community assembly processes in shaping the β-diversity of intertidal microbial communities.

**Fig 5:**
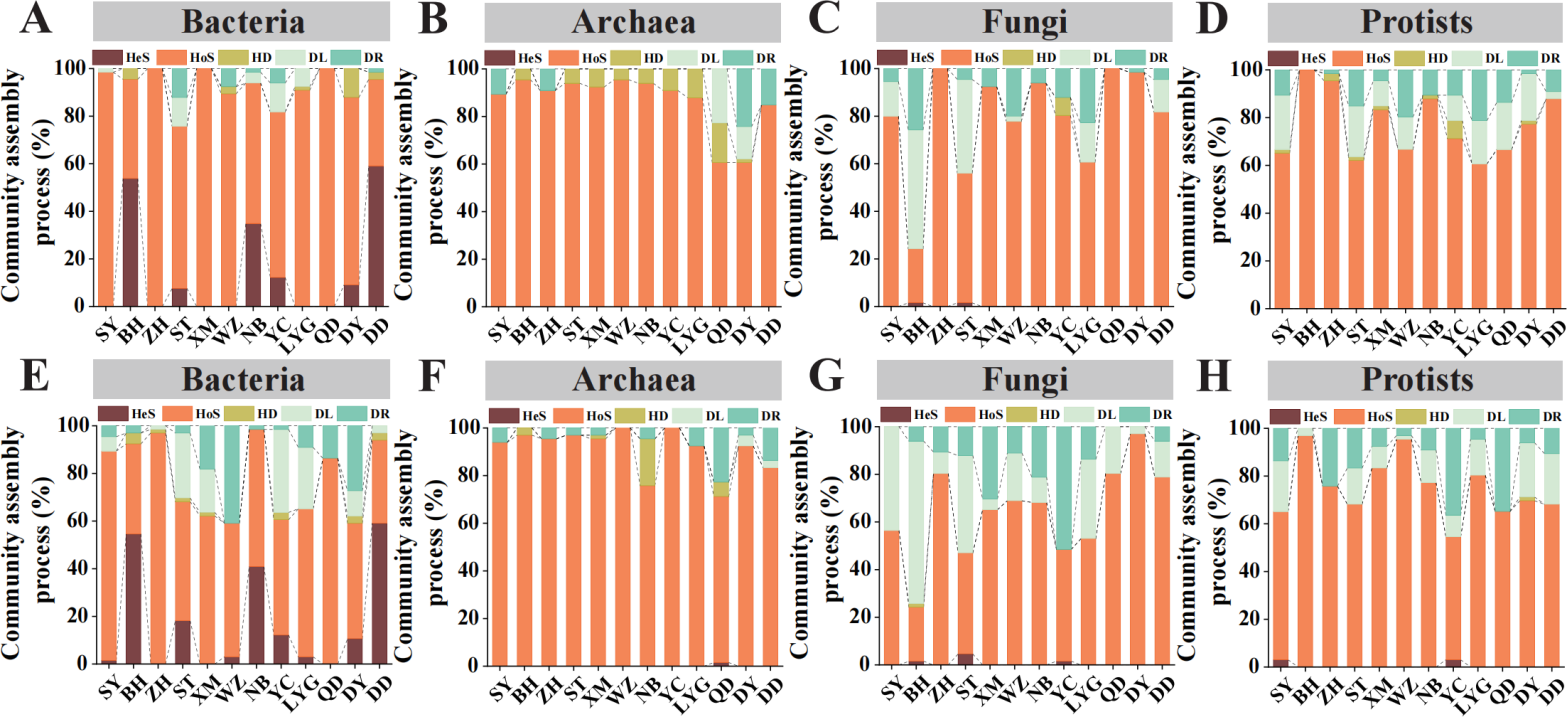
Relative contributions of local community assembly processes for different microbial domains. (**A–D**) Relative contributions of different ecological processes to community assembly using null models controlling for γ-diversity. (**E–H**) Relative contributions of different ecological processes to community assembly using null models controlling for regional species pool. Ecological processes including heterogeneous selection (HeS), homogeneous selection (HoS), homogenizing dispersal (HD), dispersal limitation (DL), and drift (DR) were quantified.

To further quantify the contribution of local community assembly process in shaping microbial β-diversity, a phylogeny-based null model approach was employed (Fig. 5). Similar results were observed when controlling for the differences in γ-diversity and the regional species pool for null models. Comparatively, fungi and protists were found with much higher ratios of dispersal limitation and drift than bacteria and archaea. For all microbial domains, homogeneous selection was the major local community assembly process mediating microbial β-diversity, though with a few exceptions. For instance, heterogeneous selection (54.5% and 59.1%) outperformed other processes for the bacterial assemblages at 21° and 39° N (Fig. 5A and E). For fungi, dispersal limitation accounted for 50.0%, 39.4%, and 16.6% of the local community assembly at 21°, 23°, and 34° N, respectively (Fig. 5C and G). These exceptional observations were generally consistent with what was observed for the patterns of β-deviations.

### Linking environmental heterogeneity with microbial β-diversity

As revealed above, environmental selection, more specifically homogeneous selection, was the major local community assembly process in shaping the microbial β-diversity. A variety of environmental variables were therefore quantified to explore the relationship between environmental heterogeneity and microbial β-diversity. For each pair of samples, the Euclidean distances of 11 environmental factors were calculated as a numerical form to represent environmental heterogeneity. Significant associations between environmental heterogeneity and β-diversity were observed for all microbial domains (Fig. S3). Consistent with the relatively lower contribution of environmental selection (Fig. 5C and G), fungi (*s* = 0.0101, *P* < 0.001) were the least affected by environmental heterogeneity among the four microbial domains (Fig. S3C). Furthermore, a significant though relatively weak association was identified between latitude and NH_4_^+^-N, NO_2_-N, and SO_4_^2−^ (Fig. S4). To further investigate the relationship between microbial dynamics and environmental variables, db-RDA using Bray–Curtis dissimilarities was performed. The results indicated that environmental variables significantly shaped microbial community compositions, with varied levels of explanatory power across different microbial groups (Fig. S5). Specifically, archaeal communities exhibited the strongest association with environmental variables (*R*² = 0.433, *P* = 0.001), primarily driven by moisture, salinity, and SO₄²⁻ (Fig. S5B). Bacterial communities were largely structured by salinity, NO₃⁻-N, and NH₄^+^-N (*R*² = 0.317, *P* = 0.001) (Fig. S5A). For fungal communities, moisture emerged as the most influential factor, with NO₂⁻-N also contributing significantly (*R*² = 0.200, *P* = 0.001) (Fig. S5C). Protist communities were shaped by salinity, SO₄²⁻, and moisture, though the total variance explained was lower than that for other groups (*R*² = 0.199, *P* = 0.001) (Fig. S5D). The complex interactions among these factors may lead to different ecological responses of various taxa under similar environmental conditions. Synthesizing the above information, we demonstrated that environmental heterogeneity along the latitudes was an important factor influencing the β-diversity of microbial communities.

## DISCUSSION

Disentangling the drivers and mechanisms mediating the biodiversity patterns across spatial and temporal scales is a crucial issue in ecology (53–55). One of the most prominent biological patterns is the increase in species varieties from the poles to the equator, often known as the latitudinal diversity gradient (LDG) (3, 4). The gradient patterns of community diversity along latitudes have been observed in both terrestrial and marine realms (3). Macrobes and microbes exhibit different α-diversity patterns along the latitudes (56–58). Multiple mechanisms and drivers have been proposed to be responsible for LDG, such as geographic area (59, 60), environmental stability (61–63), and historical effects (64, 65). As the undividable component of biodiversity, it remains less investigated how the β-diversity changes with latitudes and what ecological mechanism drives such patterns, especially for microbial communities—the “unseen majority” in the Earth’s biosphere (5).

As expected, divergent latitudinal diversity patterns were observed for different microbial domains in the intertidal wetlands, on the basis of highly differing biological and ecological features at the domain level. Typical LDG patterns (decreased α-diversity with increasing latitude) were observed for bacterial and archaeal communities, whereas such a pattern was opposite for fungal communities and nonexistent for protist communities. For bacterial and archaeal communities, higher diversity at lower latitudes aligns with patterns in terrestrial and marine ecosystems, driven by greater resource availability and stable temperatures (66, 67). Notably, the observed pattern for fungi was consistent with a few recent studies showing that fungal richness increases with increasing latitude (68, 69). Compared to prokaryotes and fungi, protists are much larger in body size and have the ability to actively move around, sheltering from temporal environmental disturbances. Such properties of protists allow them to be less affected by environmental disturbance and may result in weaker LDG patterns. Overall, this suggests that microbes may not always follow LDG patterns, though it is a well-recognized pattern in ecology.

Unlike α-diversity, the patterns of microbial β-diversity were highly disordered. Latitudinal patterns could not be observed, except for fungal communities. Such dynamic patterns disagreed with several previous findings for macrobes (13, 70), but aligned with recent studies for microbes (15, 71). Notably, different microbial domains were found with completely different β-variations along latitudes. Although bacteria and archaea were found with similar gradient patterns in α-diversity, their β-diversity patterns differed dramatically, demonstrating different ecological processes underneath. This also suggested that the dynamic intertidal zones may have exerted different effects on the compositional variations of different microbial domains.

The β-diversity in ecology describes the between-site differences of biological communities and is intimately linked with α- and γ-diversity, though such links are usually not reflected in the numeric expression (e.g., Bray–Curtis dissimilarity) (72–74). Theoretically, β-diversity shall be strongly affected by γ-diversity (or the regional species pool) at the same sampling depth (i.e., constraining the α-diversity). Previous studies have shown that changes in β-diversity are simply caused by changes in γ-diversity alone, and invoking local community assembly mechanisms does not help to explain the global latitudinal and elevational patterns in plant β-diversity (13, 75). Here, our study showed that γ-diversity was strongly and significantly associated with the latitudinal patterns of bacterial and archaeal β-diversity, but not with fungal and protist communities. Such different effects of γ-diversity on microbial β-diversity might be attributed to their specific biological characteristics, such as body size (25, 76) and genome size (77, 78). For instance, the larger body size and genome size of fungi and protists may enhance their resilience to environmental changes in intertides, resulting in enhanced local community assembly processes (e.g., drift and dispersal limitation) and less influence by the regional species pool compared to bacteria and archaea.

In addition to the regional species pool, local community assembly may also critically affect the latitudinal variation in β-diversity (15, 79, 80). Deterministic (e.g., homogeneous and heterogeneous selection) and stochastic processes (e.g., dispersal limitation, homogeneous dispersal, and drift) collectively influence the β-diversity of microbial communities (16, 20, 81). The effects of different processes may greatly vary by ecosystem types, microbial trophic levels, and spatial scales (2, 82). For example, the relative importance of stochastic processes varies by trophic levels, with the role of dispersal limitation diminishing and the role of drift increasing in the order of bacteria, fungi, protists, and soil animals (83). Similar ecosystems tend to acquire similar microbial community assembly mechanisms, but sometimes with different conclusions (84). In this study, the β-diversity of all four microbial domains significantly deviated from null expectations, either when controlling for the γ-diversity or the regional species pool, suggesting that local community assembly processes played important roles in mediating the latitudinal β-variations. Among these, homogeneous selection, via which the community compositions become more similar (16, 85), was the dominant process for intertidal microbiomes in most sampling sites, especially for archaeal communities. Noticeably, though dominated by homogeneous selection, the involvement of different processes resulted in deviated β-diversity of different microbial domains. For instance, heterogeneous selection, dispersal limitation, and drift, respectively, modulated the effects of regional species pool on the β-diversity of bacteria, fungi, and protists. Such differences shall be attributed to the domain-specific functional traits of microorganisms. For example, the higher contribution of dispersal limitation on fungi may result from their relatively larger propagule sizes and more substrate-specific growth requirements, limiting their potential for long-distance dispersal and ecological homogenization (25). For protists, their ability to actively move around plus passive movements can lead to enhanced degrees of drift (86). In contrast, the smaller body size, higher population densities, and greater metabolic plasticity of bacteria and archaea likely facilitate wider dispersal and generalist lifestyles, enhancing their responsiveness to homogenizing selective pressures (26, 87). Therefore, although shown with similar β-deviation patterns, the underlying ecological mechanisms may differ dramatically, demonstrating the necessity to quantify the contributions of different ecological processes in mediating microbial communities (20, 85).

Closely associated with local community assembly are environmental conditions. A recent study has demonstrated decreased stochastic processes under high environmental stress (88). In this study, the relationship between environmental heterogeneity and microbial β-diversity was also analyzed. High environmental heterogeneity tends to increase available niche space and provide shelter and refuge during periods of unfavorable environmental conditions and climate change, allowing for the coexistence of a greater number of species, thereby increasing microbial β-diversity (89, 90). Here, a clear association between environmental heterogeneity and latitude was found, showing increasingly varied environmental conditions with increasing latitudes in the intertidal zones. Such results suggest that environmental heterogeneity also serves as an important factor influencing the pattern of β-diversity along the latitudes.

**Fig 6:**
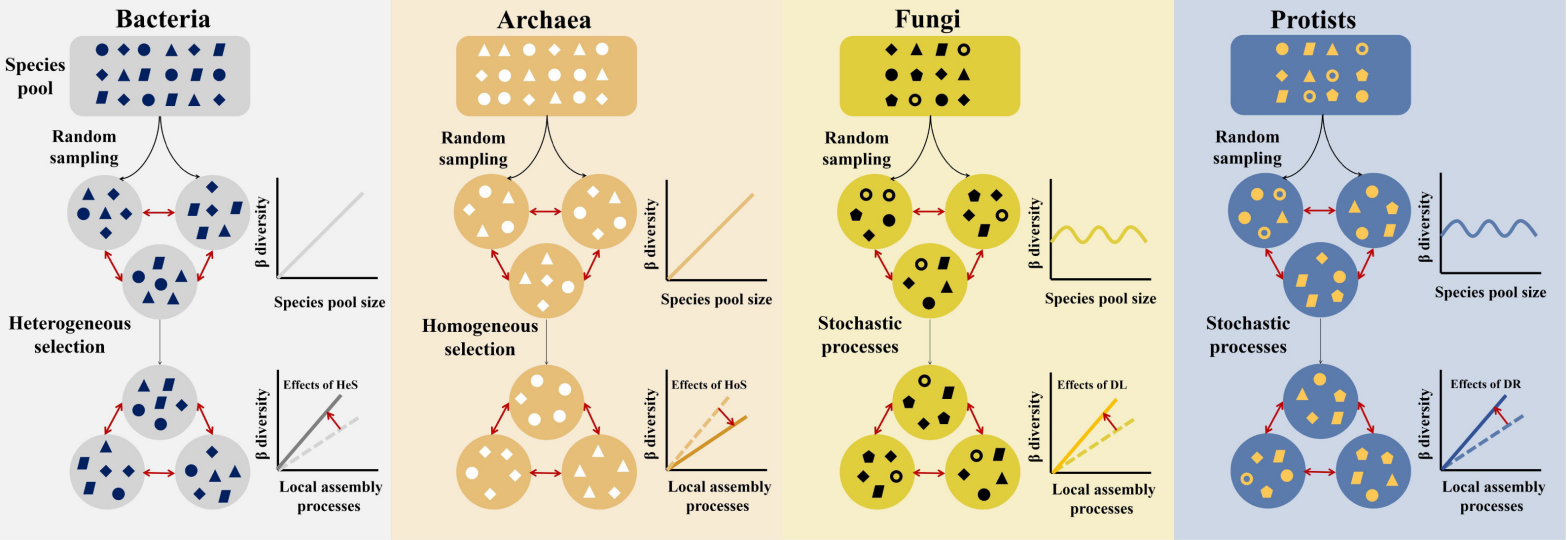
A conceptual framework describing the drivers of latitudinal β-variations for different microbial domains. Both the effects of species pools and local community assembly processes were considered. The β-diversity of bacterial and archaeal communities was influenced by both regional species pools and local community assembly, whereas fungi and protists were influenced by local community assembly. In the figure, rectangles represent regional species pools, and circles with geometric symbols represent local communities consisting of a subset of the regional species pool. Geometric symbols within circles represent different taxa; their number and variety indicate taxonomic richness and community variation. Inset plots show deviations of observed β-diversity from null expectations, with red arrows marking the direction of deviation. Ecological processes, including heterogeneous selection (HeS), homogeneous selection (HoS), dispersal limitation (DL), and drift (DR) are represented.

In this study, we also present a conceptual model that synthesizes the key findings of this study and illustrates the mechanisms driving the latitudinal β-diversity variations across microbial domains (Fig. 6). Specifically, both bacterial and archaeal communities are strongly influenced by both regional species pool and local community assembly processes. For bacteria, the relatively high β-diversity is largely driven by heterogeneous selection, whereas archaeal β-diversity remains relatively low due to the predominance of homogeneous selection. In contrast, fungi and protists are influenced by local community assembly processes, such as dispersal limitation and drift, resulting in high β-diversity. This model illustrates how the regional species pool and local community processes shape microbial communities along latitudinal gradients, emphasizing the need to consider domain-specific mechanisms in microbial biogeography.

In conclusion, this study comparatively examined the ecological drivers shaping the β-diversity across different microbial domains along a broad climatic gradient in intertidal mudflats. Different microbial domains exhibited diverse β-diversity patterns along the latitudinal gradient, shaped by different contributions of regional species pools and local community assembly. While bacterial and archaeal communities were influenced by both regional species pool and local community assembly, fungi and protists were mainly driven by local community assembly processes. Although homogeneous selection dominated across all microbial domains, the contributions of other ecological processes underscore domain-specific community assembly. This study provides a clearer understanding of the mechanisms that drive latitudinal β-variations for different intertidal microbiomes. As current studies mainly focus on the taxonomic variations of microbial communities, ignoring the functional traits carried by different groups, future studies may explore how different dimensions of biodiversity, especially functional diversity, contribute to the community assembly of microbial communities, with the effort to develop a unified framework across different biological domains/kingdoms (91).

## Data Availability

The sequencing data generated in this study have been deposited at the NCBI SRA portal under project ID PRJNA957716.

## SUPPLEMENTAL MATERIAL

Supplemental Figures msystems.01777-25-s0001.docx Figures S1 to S6.

Supplemental Tables msystems.01777-25-s0002.xlsb Tables S1 to S3.

Table S4A msystems.01777-25-s0003.xlsb ASV information for bacteria.

Table S4B msystems.01777-25-s0004.xlsb ASV information for archaea.

Table S4C msystems.01777-25-s0005.xlsb ASV information for fungi.

Table S4D msystems.01777-25-s0006.xlsb ASV information for protists.

Captions msystems.01777-25-s0007.docx Captions for supplemental tables.

ASM does not own the copyrights to Supplemental Material that may be linked to, or accessed through, an article. The authors have granted ASM a non-exclusive, world-wide license to publish the Supplemental Material files. Please contact the corresponding author directly for reuse.
