# Supplementary material for: Disentangling drivers of cross-domain microbial β-variations in intertidal mudflats
Source: mSystems. 2026 Feb 26;11(3):e01777-25. doi: 10.1128/msystems.01777-25 (PMC13011393; doi:10.1128/msystems.01777-25)
Supplement: Supplemental Figures — Figures S1 to S6. [file msystems.01777-25-s0001.docx]

**Supplementary Figures**

**
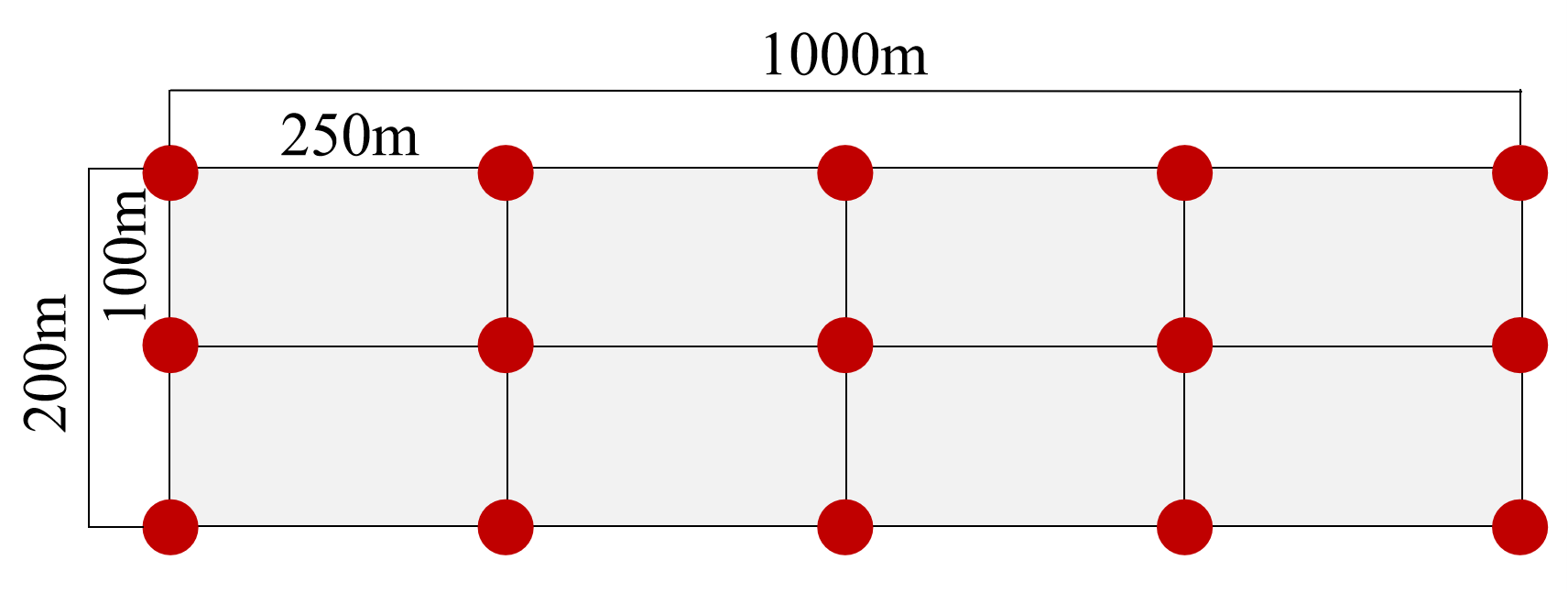
**

**Supplementary Figure 1 Schematic diagram of the sampling design within each region.** Fifteen sampling points (red circles) were evenly distributed across a 1000 m × 200 m area, with 250 m spacing between adjacent points along the coastline and 100 m spacing toward the ocean. At each location, five surface sediment cores were collected and homogenized into one composite sample.


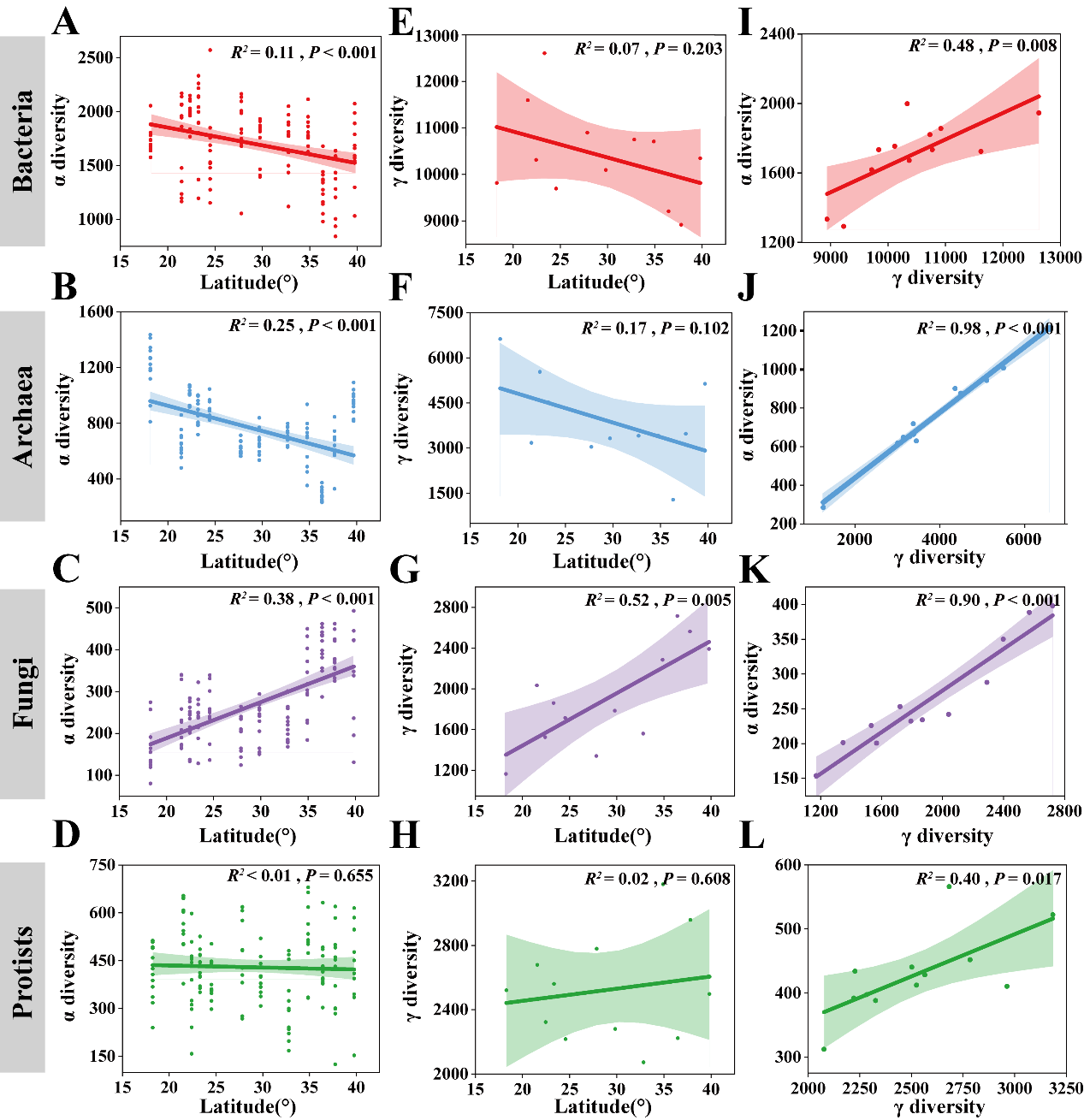
**Supplementary Figure 2 Diversity patterns of different microbial domains along the latitudinal gradient and the relationship between α- and** **γ-diversity. (A-D)** The α-diversity (the number of observed ASVs) patterns of different microbial domains along the latitudes. **(E-H)** The γ-diversity patterns of different microbial domains along the latitudes. **(I-L)** The relationship between α- and γ-diversity of different microbial domains.

**
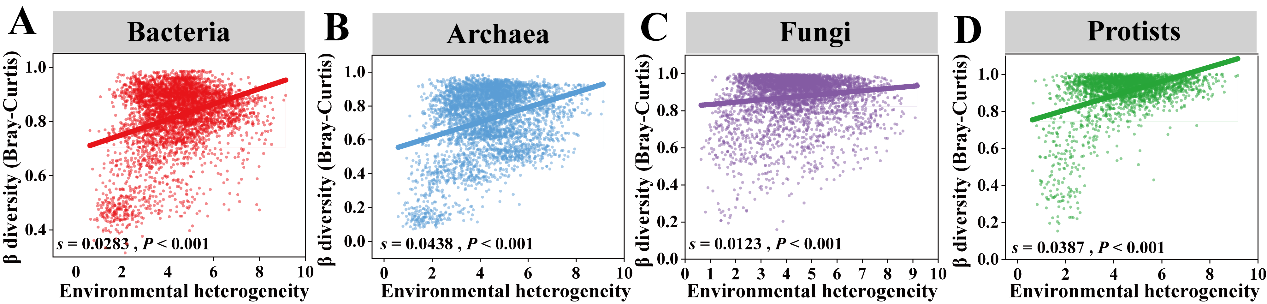
Supplementary Figure 3 Linking β-diversity of different microbial domains to environmental heterogeneity.** The environmental heterogeneity between different samples was calculated as the Euclidean distances based on normalized environmental variables. Different colors represent different microbial domains, including bacteria (A), archaea (B), fungi (C), and protists (D).


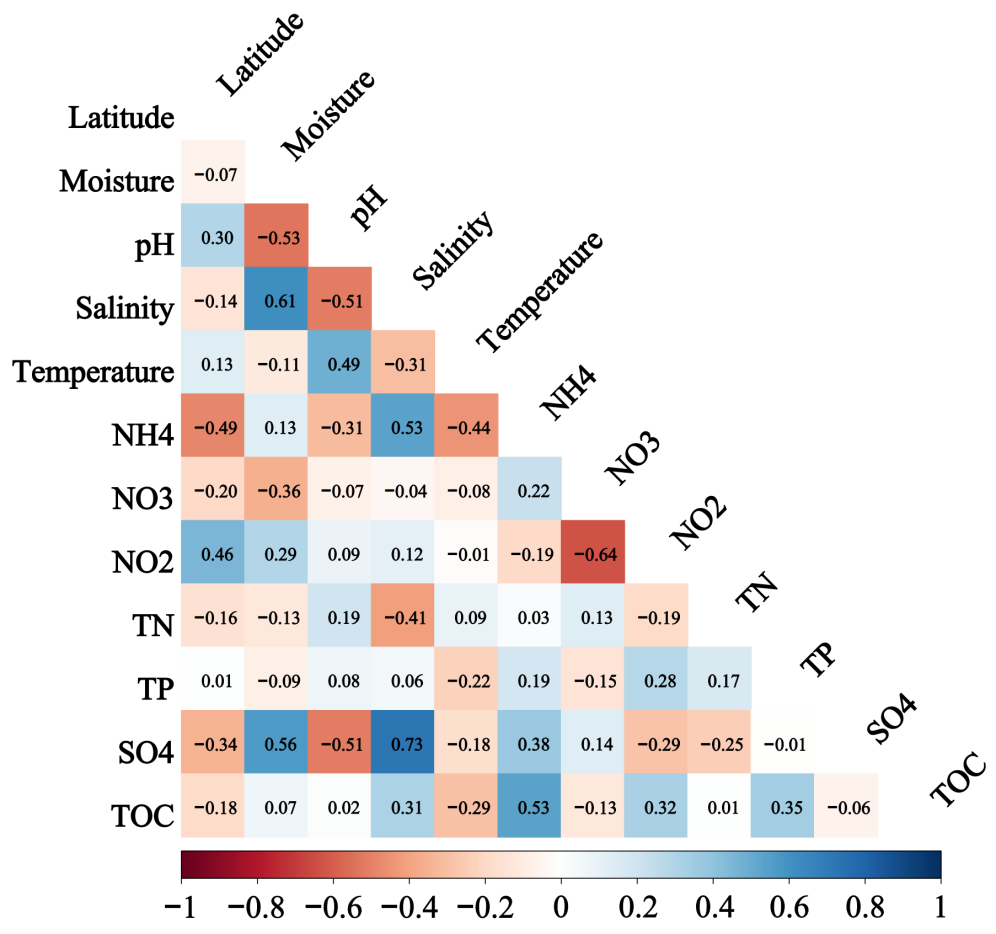


**Supplementary Figure 4 The relationship between latitudes and environmental variables.** The correlation relationship between latitude and environmental variables. The color gradient represents Pearson’s correlation coefficients, with blue indicating positive correlations and red indicating negative correlations.

**
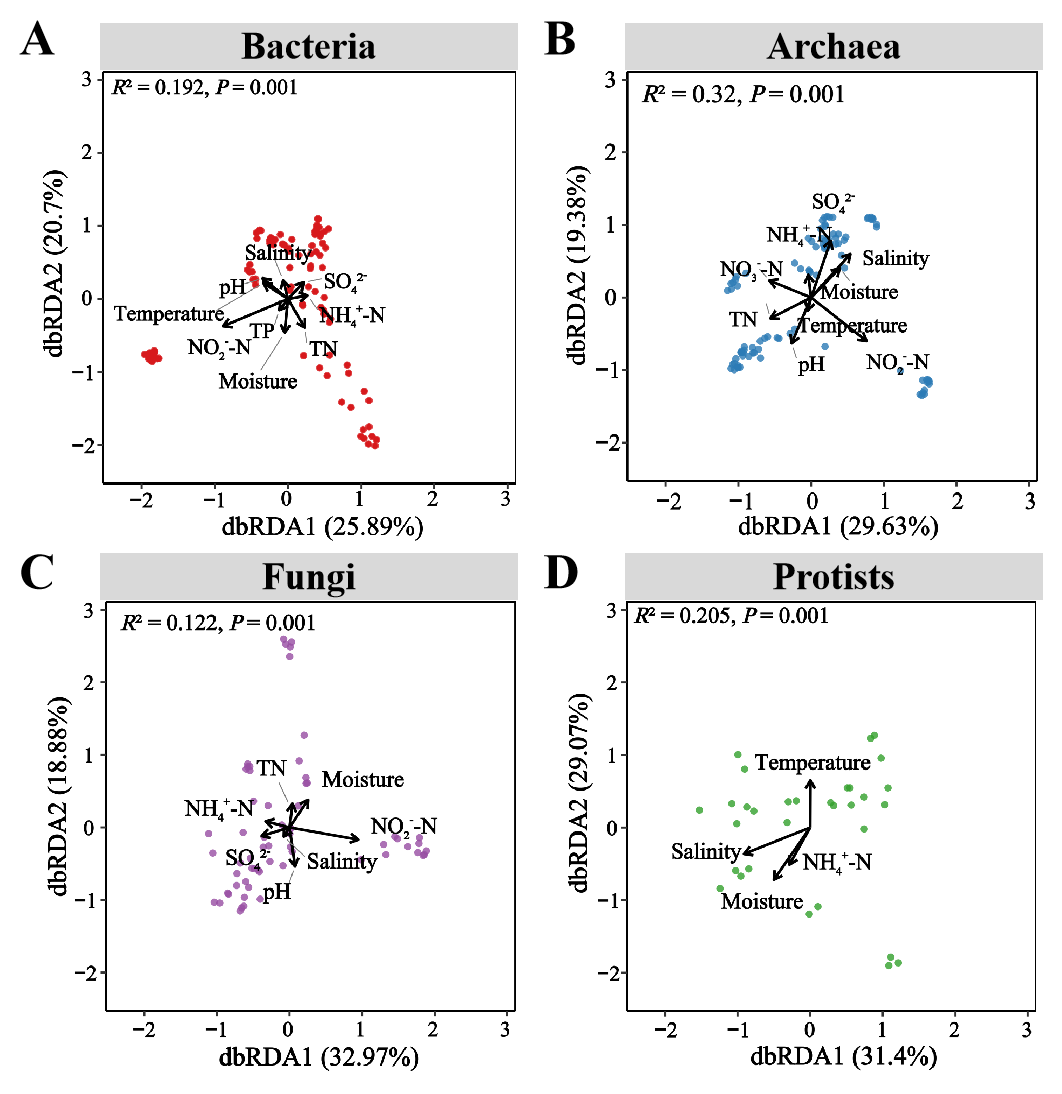
Supplementary Figure 5 Constrained db-RDA showing the associations between environmental variables and community compositions for different microbial domains, including bacteria (A), archaea (B), fungi (C), and protists (D).** The Bray-Curtis dissimilarity was used to measure the compositional variations of microbial communities. The relationships between compositional variations and environmental variables were examined by db-RDA, followed by forward selection to retain significant explanatory variables. In the figure, each point represents a microbial community sample, and the arrows indicate the direction and relative strength of the environmental gradients. The percentage of variance explained by the first two axes is indicated along the axes.

**
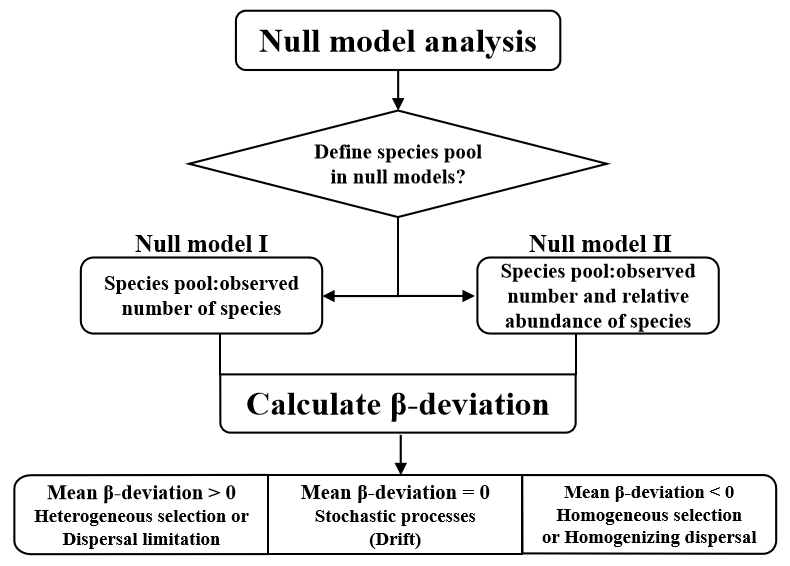
**

**Supplementary Figure 6 A conceptual framework for β-deviation analysis based on null models.** Null model I defines the species pool as the observed number of species (γ-diversity), while null model II incorporates both observed species richness and relative abundance (regional species pool). The β-deviation was calculated as the standardized difference between observed and expected β-diversity.
