# Supplementary material for: Disentangling drivers of cross-domain microbial β-variations in intertidal mudflats
Source: mSystems. 2026 Feb 26;11(3):e01777-25. doi: 10.1128/msystems.01777-25 (PMC13011393; doi:10.1128/msystems.01777-25)
Supplement: Captions — for supplemental tables. [file msystems.01777-25-s0007.docx]

**Description of Additional Supplementary Files**

**Supplementary Table 1:** Geographical Parameters and Tidal Characteristics of the sampling sites.

**Supplementary Table 2:** Measured environmental parameters of the sampling sites. NH_4_^+^-N: Ammonia-nitrogen; NO_2_^-^-N: Nitrite nitrogen; NO_3_^-^-N: Nitrate-nitrogen; TN: Total nitrogen; TP: Total phosphorus; SO_4_^-^: water-soluble sulfate; TOC: Total organic carbon.

**Supplementary Table 3A:** Summary of sequencing data of bacteria.

**Supplementary Table 3B:** Summary of sequencing data of archaea.

**Supplementary Table 3C:** Summary of sequencing data of fungi.

**Supplementary Table 3D:** Summary of sequencing data of protists.

**Supplementary Table 4A:** Amplicon sequence varians (ASVs) information of bacteria.

**Supplementary Table 4B:** Amplicon sequence varians (ASVs) information of archaea.

**Supplementary Table 4C:** Amplicon sequence varians (ASVs) information of fungi.

**Supplementary Table 4D:** Amplicon sequence varians (ASVs) information of protists.
